# Development of a method to involve patients in the determination and appraisal of outcomes in clinical trials

**DOI:** 10.1186/1745-6215-16-S1-P15

**Published:** 2015-05-29

**Authors:** Charlotte M W Gaasterland, Marijke C Jansen-van der Weide, Johanna H van der Lee

**Affiliations:** 1 Pediatric Clinical Research Office Woman-Child Center, Academic Medical Center, University of Amsterdam, Meibergdreef 9, 1105 AZ Amsterdam, The Netherlands

## Introduction

Involving patients in the early stages of trial design may be relevant from an ethical point of view, since the patients are those who are most likely to benefit from the research. Also, the quality of the research may benefit from the involvement of patients, who may suggest research questions and outcome measures that are relevant to them, as well as procedures that may lead to better recruitment and retainment. In drug trials, for instance, it may be relevant to ask patients what they expect of a new medication and what they hope the effects are. The instruments used to measure these effects are of pivotal importance for the result of the trial and the subsequent decision-making. In rare diseases the choice of outcomes, and the relative value of several outcomes in a possible composite outcome, is even more important. However, until now there is no standard method to involve patients in the choice and appraisal of outcomes. Therefore, we want to develop a methodology to involve patients in the decisions about relative weights of outcomes in the trial design stage.

## Methods

First, we have performed a literature review on patient involvement in research design, and consulted professional patient representatives. Based on these meetings and the literature, we have developed a draft method to involve patients in the determination and weighing of outcome measures. We will elicit feedback from professional stakeholders, e.g. clinical researchers, pharmaceutical companies, regulators, and from a group of patient representatives, which will be used to improve our methods. Subsequently, we will test the methods by applying them in the developmental stage of an actual trial. All contacts with external parties are arranged through the ASTERIX consortium (http://www.asterix-fp7.eu).

Results will become available in 2015.

